# Occurrence investigation of perfluorinated compounds in surface water from East Lake (Wuhan, China) upon rapid and selective magnetic solid-phase extraction

**DOI:** 10.1038/srep38633

**Published:** 2016-12-14

**Authors:** Yusun Zhou, Yun Tao, Huarong Li, Tingting Zhou, Tao Jing, Yikai Zhou, Surong Mei

**Affiliations:** 1 Key Laboratory of Environment and Health, Ministry of Education & Ministry of Environmental Protection, and State Key Laboratory of Environment Health (Incubation), School of Public Health, Tongji Medical College, Huazhong University of Science and Technology, Wuhan 430030, Hubei, China; 2 Department of Clinical Laboratory, The Affiliated Hospital of Qingdao University, Qingdao 266003, Shandong, China; 3 Department of Pharmacy, Jingzhou Hospital, Tongji Medical College, Huazhong University of Science and Technology, Jingzhou 434020, Hubei, China

## Abstract

Using a novel magnetic nanocomposite as adsorbent, a convenient and effective magnetic solid-phase extraction (MSPE) procedure was established for selective separation and concentration of nine perfluorinated compounds (PFCs) in surface water sample. Then an ultra high-performance liquid chromatography-tandem mass spectrometry (UPLC-MS/MS) system was employed for detection of PFCs. Good linearity of the developed analytical method was in the range of 0.5–100 ng L^−1^ with R^2^ > 0.9917, and the limits of detection (LODs) ranged from 0.029 to 0.099 ng L^−1^. At three fortified concentrations of 0.5, 5 and 50 ng L^−1^, the spiked recoveries of PFCs were in the range of 90.05–106.67% with RSDs < 12.62% (n = 3). The proposed analytical method was applied for determination of PFCs in surface water from East Lake (Wuhan, China). The total concentrations of nine PFCs ranged from 30.12 to 125.35 ng L^−1^, with perfluorooctane sulfonate and perfluoroctanoic acid as the most prevalent PFCs, and the greatest concentrations of PFCs were observed in Niuchao lakelet. The concentrations of the PFCs (C ≥ 11) were mostly less than the limits of quantification (LOQs), attributed to the possibility that the more hydrophobic long-chain PFCs are potential to accumulate in sediment and aquatic biota.

Perfluorinated compounds (PFCs) are a group of anthropogenic organofluorine chemicals, usually existing in two persistent forms of perfluoroalkyl sulfonates (PFSAs) and perfluoroalkyl carboxylates (PFCAs), and their unique properties have made them useful in a number of industrial and commercial applications for more than 50 years^1^,^2^. In relation to human exposure to PFCs, several different pathways are suggested, including food consumption, drinking water and dust inhalation^3^,^4^,^5^. PFCs mainly accumulate in blood, liver and kidney of living organisms due to their high water-solubility, in comparison with other well-studied organic pollutants that are of adipose-accumulative toxicity^6^,^7^,^8^. Animal-related poison experiments and human epidemiological studies have revealed PFC adverse effects, such as hepatotoxicity, immunotoxicity, developmental and reproductive toxicity, hormonal effects and carcinogenic potency^9^,^10^,^11^,^12^. In recent years, PFCs have been frequently detected in various environmental waters and strongly proved to accumulate in aquatic biotas, which makes the aquatic ecosystem as an important medium for PFC transport^13^,^14^. Therefore, supervising the occurrence of PFCs in aquatic environment is of great importance to reduce human exposure and eliminate adverse effects.

PFCs are typically present at ng L^−1^ level in environmental waters, and consequently, the direct mass spectrometry analysis is difficult without separation and pre-concentration. Solid-phase extraction (SPE) is a routine method for determination of trace PFCs in water sample^13^,^15^,^16^. However, traditional SPE is not applicable to rapid pretreatment of large-volume water sample. For the past few years, an effective magnetic SPE (MSPE) technique has been established with magnetic material used as adsorbent^17^,^18^,^19^. The magnetic adsorbent was dispersed in water sample, and PFCs were extracted from water matrices in a short time. After that, the adsorbent was separated easily by an external magnetic field, and the adsorbed PFCs were eluted for further analysis, which made the MSPE procedure greatly simplified and more suitable for rapid detection of PFCs in environmental waters. Unfortunately, the environmental water matrices are complex and the matrix co-extraction by conventional MSPE with non-selective magnetic material as adsorbent may occur, which has a negative influence on PFC extraction and detection. Therefore, for the accurate quantification of PFCs in water sample, a selective and effective magnetic adsorbent should be highly required.

A fluorine-fluorine (F-F) interaction was depicted by Gladysz and Curran as a noncovalent force that occurs between a fluorous medium and a fluorous portion of a molecule^20^. Lately, Oleschuk deduced that the F-F interaction may be a special instantaneous dipole-dipole force^21^. Although the fundamental of the F-F interaction remains to be elucidated, researchers have developed a number of ways to exploit this attraction for chemical purification and separation^22^,^23^. Most recently, combining the selective recognition ability of F-F interaction and rapid magnetic separation property of Fe_3_O_4_ nanoparticles (NPs), two useful magnetite materials were prepared for extraction and pre-concentration of PFCs in surface water samples^24^,^25^. But in these two studies, limited sorption ability (<0.4 μg g^−1^) was obtained with the magnetic adsorbents only functionalized by fluorous groups. In our previous study, an effective magnetic nanocomposite (Fe_3_O_4_@SiO_2_-NH_2_&F_13_) functionalized with both amino group and octyl-perfluorinated chain has been prepared^26^. Compared with the two up-mentioned magnetic materials, the composite exhibited good affinity and selectivity for PFCs and showed a high adsorption capacity and a fast sorption kinetics.

Herein, in this study, using the prepared Fe_3_O_4_@SiO_2_-NH_2_&F_13_ as magnetic adsorbent, a convenient, rapid and effective MSPE procedure was established for selective extraction of PFCs in surface water from East Lake (Wuhan, China). An ultra high-performance liquid chromatography-tandem mass spectrometry (UPLC-MS/MS) system was used for separation and detection of the concentrated PFCs. Moreover, the occurrence and distribution of multiple PFCs in the lake water were also discussed, which would provide detail information for further pollution investigation of PFCs in East Lake.

## Results and Discussion

### Establishment of MSPE procedure

In this study, water samples were analyzed for nine PFCs, including perfluorohexane sulfonate (PFHxS), perfluorooctane sulfonate (PFOS), perfluoroheptanoic acid (PFHpA), perfluorooctanoic acid (PFOA), perfluorononanoic acid (PFNA), perfluorodecanoic acid (PFDA), perfluoroundecanoic acid (PFUnDA), perfluorododecanoic acid (PFDoDA) and perfluorotetradecanoic acid (PFTA). Initially, the influences of several parameters on MSPE efficiency were evaluated in terms of PFC extraction recoveries, such as the elution solution, adsorbent amount, washing solution and sorption time. Our previous study has verified the low water pH value was beneficial for PFC adsorption on the prepared Fe_3_O_4_@SiO_2_-NH_2_&F_13_^26^. Thus, the water pH value of 3 was defined and applied for MSPE optimization and further sample preparation in this study. The optimization experiments were conducted using 500 mL of nine-PFC aqueous solution with a spiked concentration at 20 ng L^−1^ level.

**Figure 1:**
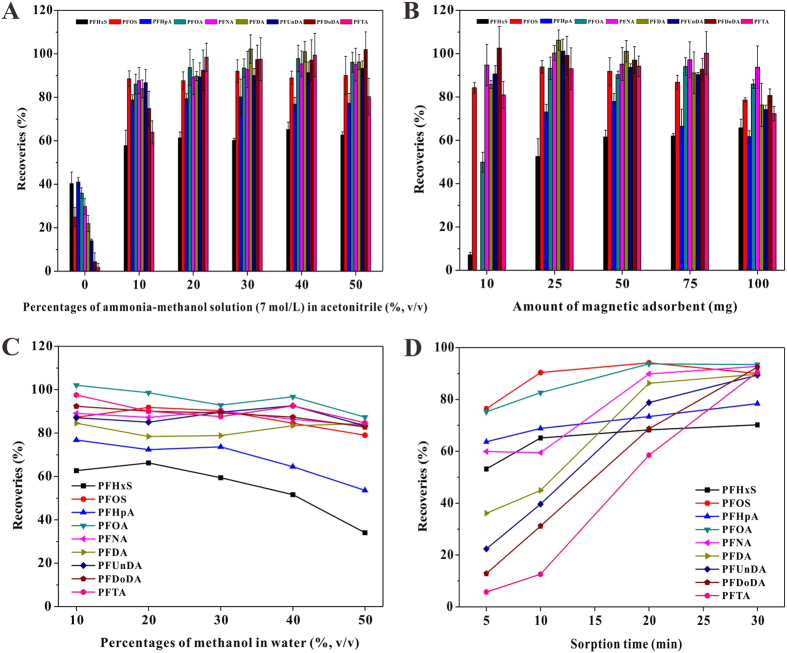
The effects of (**A**) elution solution, (**B**) adsorbent amount, (**C**) washing solution and (**D**) sorption time on the extraction recoveries of PFCs in 500 mL of purified water with Fe_3_O_4_@SiO_2_-NH_2_&F_13_ as MSPE adsorbent.

PFCs could be eluted from Fe_3_O_4_@SiO_2_-NH_2_&F_13_ with the help of a suitable solution disrupting the affinity of magnetic adsorbent for target analytes. Initially, 50 mg of the adsorbent was used for PFC extraction from aqueous solution, and the effects of a series of organic solvents with different polarity (methanol >acetonitrile ≈ ethanol >acetone >chloroform >ethyl acetate >dichloromethane) on PFC recoveries were investigated. The results showed that only acetonitrile had a certain of eluting effect, but less than 40% of PFCs were recovered from the magnetic adsorbent, and the recoveries decreased with the increasing C-F length of PFCs (Fig. 1A). Subsequently, acetonitrile with several different percentages of ammonia-methanol solution (7 mol L^−1^) were used as elution solutions. Our previous study has stated the interactions between the prepared magnetic adsorbent and the PFC molecule^26^, which include electrostatic and F-F interaction, and a conclusion that the selective F-F interaction is promoted by the electrostatic attraction has also been drawn. In alkaline solution, the deprotonated −NH_2_ on Fe_3_O_4_@SiO_2_-NH_2_&F_13_ surface hardly attracts any anionic –COOH and –SO_3_H of PFC molecule^27^, consequently weakening the F-F interaction of the adsorbent for C-F chain in PFC molecule, which results in the desorption of the adsorbed PFCs from the magnetic adsorbent. Therefore, as shown in Fig. 1A, alkaline solution had improved PFC recoveries significantly, and especially, most PFCs were eluted from Fe_3_O_4_@SiO_2_-NH_2_&F_13_ when acetonitrile: ammonia-methanol solution (7: 3, v/v) was used as the elution solution.

The prepared Fe_3_O_4_@SiO_2_-NH_2_&F_13_ exhibited good selectivity and high adsorption capacity for PFCs by electrostatic and F-F interaction with the addition of size exclusion effect^26^. Although higher amount of the material might be good for more PFC adsorption, the elution of PFCs adsorbed on the material should take more desorption time or eluent consumption^25^. Thus, this study used 10–100 mg of the magnetic adsorbent for PFC extraction and then separated the adsorbent by an external magnetic field. PFCs bound on the adsorbent were eluted by acetonitrile: ammonia-methanol solution (7: 3, v/v). It can be seen in Fig. 1B that higher adsorbent amounts could distinctly enhance PFC extraction efficiency, especially for PFHxS and PFHpA, but PFC recoveries had no obvious increase with the adsorbent amount more than 50 mg, and even decreased when the amount was 100 mg. Herein, 50 mg of magnetic material was used as the effective MSPE adsorbent for PFC extraction from water sample.

In traditional SPE procedure for PFC pre-concentration in water sample, neutral or acidic purified water with more or less methanol (5–50%, v/v) was often used for removal of matrix compounds^15^,^28^,^29^. Thus, in this study, neutral water with several percentages of methanol were used as washing solution for removal of interference matrices non-selectively adsorbed on the adsorbent during PFC extraction from water sample. The effects of methanol contents in water on PFC recoveries were shown in Fig. 1C, and it can be seen that the presence of methanol in washing solution had hardly any influence on PFC recoveries. Only when the proportion of methanol in water was higher than 30%, the recoveries of PFHxS and PFHpA decreased. Consequently, methanol: water (3: 7, v/v) was used as the washing solution for further studies.

The rapid sorption kinetics of Fe_3_O_4_@SiO_2_-NH_2_&F_13_ for PFCs in water has been confirmed in our previous study^26^, and the adsorption equilibrium could be achieved in 5 min, which is ascribed to the characters of surface sorption taking place on silica shell where only external diffusion was involved^26^. However, PFCs are typically present at ng L^−1^ level in environmental waters that is much lower than the concentration used for sorption kinetics investigation, which reduces the mass transfer rate between analyte and adsorbent^30^. For this reason, it should take additional energy and longer sorption time to acquire an effective extraction of PFCs by the magnetic composite with its amount fixed. In this study, 50 mg of Fe_3_O_4_@SiO_2_-NH_2_&F_13_ was added into 500 mL of purified water spiked with 1 ng L^−1^ of PFCs. The extraction system was given a sonicate (200 W) process for 5 min and then kept for static sorption at room temperature. The influence of the sorption time on PFC recoveries was investigated. As can be seen in Fig. 1D, 30 min of sorption time was required for the achievement of sorption equilibrium at the trace level of PFC concentration. Therefore, a sorption time of 30 min was opted for PFC extraction from lake water sample.

At the optimized MSPE conditions, PFCs were extracted from 500 mL of purified water spiked at 0.5, 5 and 50 ng L^−1^ levels (n = 3). The PFC extraction recoveries ranged from 59.29 to 94.95% with the relative standard deviations (RSDs) lower than 9.06%. Therefore, the proposed MSPE procedure was suitable for rapid preparation of large-volume water sample.

### Development and validation of MSPE-UPLC-MS/MS method

PFCs were added into 500 mL of purified water with the final concentration at 0.5–200 ng L^−1^ levels, and then 5 ng L^−1^ of isotopic internal standards (ISs) were also added into the water. PFCs were extracted by the optimized MSPE procedure and subjected to UPLC-MS/MS analysis. The calibration curve was plotted with the PFC spiked concentration as X-axis, and the ratio of chromatographic peak area for PFC and its corresponding IS as Y-axis. The ^13^C_4_-labeled PFOS (MPFOS) and the labeled PFOA (MPFOA) were used as IS surrogates for PFSAs and PFCAs, respectively. As can be seen in Table 1, good linearity of the nine PFCs was achieved in the range of 0.5–100 ng L^−1^ with R^2^ > 0.9917.

**Figure 2:**
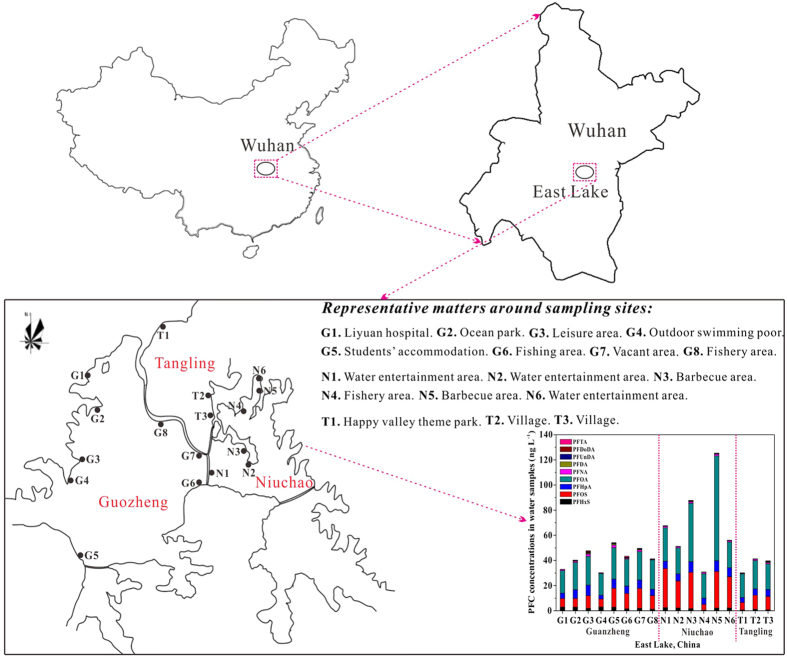
Sampling sites in surface water from East Lake and PFC concentrations in each area. (This figure was created by author Yusun Zhou, using CorelDRAW Graphics Suite X6, and the URL link is http://www.coreldraw.com/cn/product/graphic-design-software/ for CorelDRAW).

The limit of quantification (LOQ) and detection (LOD) for each PFC were determined by spiking seven replicates in the mixed lake water sample with PFCs at 1 ng L^−1^ level. The mixed water sample was prepared by mixing 1 L of water sample collected in each sampling site (Fig. 2) with each other in a polypropylene (PP) bucket, giving rise to a total water volume of about 17 L. The LOQs and LODs were defined as 10 times and 3 times the standard deviations of PFC concentrations calculated by the plotted calibration curves. It can be seen in Table 1 that the LOQs and LODs of the analytical method were in the range of 0.097–0.330 ng L^−1^ and 0.029–0.099 ng L^−1^, respectively. The high sensitivity of our proposed method made it fit for determination of trace PFCs in environmental water sample.

On the other hand, during the validation and application of the proposed MSPE-UPLC-MS/MS method, the procedural blank was executed in the same process by using 500 mL of purified water, to make sure that the background contamination signals were less than LOQs. In fact, all PFCs had a field blank lower than LODs, except PFOA (<0.062–0.208 ng L^−1^).

For the validation of the developed MSPE-UPLC-MS/MS method, the accuracy and precision, expressed as the spiked recoveries and RSDs, were evaluated at three fortified concentrations of 0.5, 5 and 50 ng L^−1^ of PFCs in the mixed lake water sample and three replicates were performed at each level. For comparison, the mixed water sample was also prepared by BS ISO 25101:2009 standard method^31^ and subjected to UPLC-MS/MS for analysis.

As shown in Table 2, the spiked recoveries of PFCs extracted by Fe_3_O_4_@SiO_2_-NH_2_&F_13_ ranged from 90.05 to 106.67% with RSDs < 12.62%. Although good precision (RSDs < 10%) was obtained when PFCs were extracted by Oasis-Wax recommended by BS ISO 25101:2009, the spiked recoveries of PFCAs at 0.5 ng L^−1^ level were lower (63.74–86.75%), especially obvious for long-chain (C11–14) PFCAs and PFHpA.

**Figure 3:**
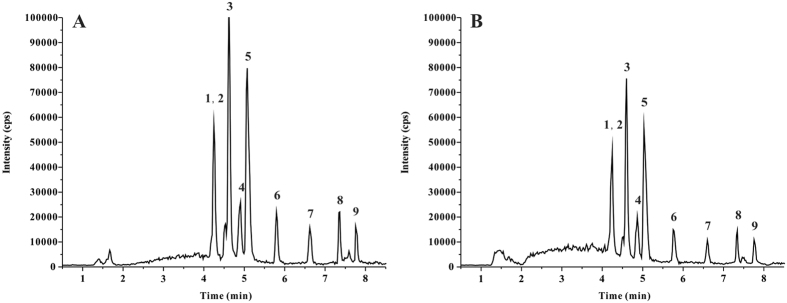
Chromatograms of the mixed water sample spiked with 5 ng L^−1^ of PFCs extracted by (**A**) Fe_3_O_4_@SiO_2_-NH_2_&F_13_ and (**B**) Oasis-Wax, respectively. (1, 2) PFHxS, PFHpA, (3) PFOA, (4) PFOS, (5) PFNA, (6) PFDA, (7) PFUnDA, (8) PFDoDA, (9) PFTA.

Figure 3 exhibited the typical chromatograms of nine PFCs extracted with Fe_3_O_4_@SiO_2_-NH_2_&F_13_ (Fig. 3A) and Oasis-Wax (Fig. 3B) from the spiked water sample. Obviously, the response signals of PFCs with Fe_3_O_4_@SiO_2_-NH_2_&F_13_ extraction were greatly enhanced, with a smooth chromatogram baseline and an effective matrix elimination. Therefore, the proposed MSPE-UPLC-MS/MS method had good accuracy and precision, attributed to the high selectivity of the used MSPE adsorbent prepared in our previous study^26^.

### Comparison with other analytical methods

The analytical performance of the MSPE-UPLC-MS/MS method proposed in this study was compared with recently published procedures for determination of PFCs in water sample, as described in Table 3. The extraction times referred in these analytical methods were different, produced by the variation of the used adsorbents. In contrast with traditional SPE technique, the MSPE with magnetic material as adsorbent made the sample preparation time greatly shortened. The extracted PFCs could be separated by UPLC system in a short time with less organic solvent consumption. Furthermore, the prepared Fe_3_O_4_@SiO_2_-NH_2_&F_13_ used as MSPE adsorbent had good selectivity and high adsorption capacity for PFCs, which afforded the proposed method a wide linearity range, a high sensitivity and a good stability in addition with a fine resistibility to water matrices. Moreover, Fe_3_O_4_@SiO_2_-NH_2_&F_13_ preparation was very convenient, environmentally friendly and easy to control, which made the composite more practical for PFC pollution monitoring in environmental waters.

### Occurrence investigation of PFCs in surface water from East Lake

The East Lake, located in Wuhan city, is the second largest urban lake in China, with a total water area of 33 km^2^ and an average depth of 2.5 m. A number of enterprises, hospitals and communities are built around the lake, which makes it very important for industrial production, life of local residents and tourist entertainment. However, during the past decades, with the rapid industrialization and urbanization, chemical pollutants were continuously discharged into the lake that has no dilution ability due to its closed shape^32^,^33^. Presently, the existence of PFOS and PFOA in East Lake has been proved by researchers^34^. Unfortunately, the spatial distribution of multiple PFCs and their composition in lake water have not been reported until now. Therefore, in this study, the occurrence of PFCs in surface water from East Lake was investigated based on our proposed MSPE-UPLC-MS/MS method.

The spatial distribution and relative composition of PFCs were summarized in Fig. 2 and Table 4. The total concentrations of nine PFCs in collected water samples ranged from 30.12 to 125.35 ng L^−1^. The Niuchao lakelet was found with the greatest concentrations of PFCs, consistent with the finding in Chen’s research^34^ in which the highest concentrations of PFOS and PFOA were detected in eastern area of the lake. The effluents flow out from manufacture plants and communities around Niuchao lakelet contributed the majority of PFC contamination. In addition, the recreational activities loaded in this area and its relative closed feature are also the important reasons for PFC pollution.

Among the nine PFCs analyzed in this study, PFOS and PFOA occurred predominantly in surface water, accounting for 15–46% and 39–67% of the total concentrations of PFCs for all the sampling sites, which was in agreement with most reports characterizing PFCs and their distribution in environmental waters. So *et al*. had pointed out that an independent source of PFOS in the nearby area might be identified according to the PFOS/PFOA ratio at the sampling site^35^. PFOS/PFOA ratios were all less than 1 (0.24–0.70), except for N1, N2 and N6 (1.04–1.21), indicating that PFOA was the dominant PFC in most areas of East Lake and PFOS contamination source maybe exist near the areas of N1, N2 and N6.

PFHxS, PFHpA and PFNA were detected in all the collected water samples with concentrations greater than the LOQs, which confirmed that the water pollution caused by these three PFCs should be also paid attentions. In most (>70%) of the water samples, the concentrations of PFUnDA, PFDoDA and PFTA were less than the LOQs, but this phenomenon could not reveal their authentic contamination for the entire ecosystem of the East Lake. A relevant research had reported that long-chain (C > 8) PFCs were potential to accumulate in sediment and aquatic biota^13^, resulting in reduced concentrations of these PFCs in surface water. Therefore, for the future studies, a comprehensive investigation is necessary by taking the occurrence of PFCs in sediments and aquatic biotas of East Lake into account.

Furthermore, nonparametric tests were carried out to reveal the correlations among the nine PFCs (Table 5). PFOS was observed to be positively correlated to PFHpA, PFOA and PFNA. The four PFCs also showed significant correlations with ΣPFCs. The positive results indicated a possibility of a common pollution source for the four PFCs in East Lake^36^. As to PFOA, a dominant PFC pollutant contributing most concentration as PFOS in lake water, was positively correlated to PFDA besides PFOS, PFHpA and PFNA. The obtained results signified more than one contamination source discharging PFOA to East Lake^37^. In addition, the long-chain (C > 8) PFCs had statistical correlations to each other, possibly mainly attributed to oxidation of telomer alcohols (FTOHs) leading to PFCAs formation^38^. It is worth noting that the short-chain PFHpA and the long-chain PFCs had significant correlations and this finding should be studied and verified in future researches.

In summary, a convenient, rapid and effective MSPE procedure was established with a novel Fe_3_O_4_@SiO_2_-NH_2_&F_13_ as magnetic adsorbent. The composite was dispersed into the water sample and PFCs could be selectively separated and concentrated in a short time (<30 min). Compared with the traditional SPE with adsorbent packed into a cartridge, the proposed MSPE significantly improved the extraction efficiency for PFCs. Furthermore, the PFCs in surface water from East Lake were accurately and sensitively determined by the proposed MSPE-UPLC-MS/MS method. The surface water had a widespread contamination of PFCs with PFOS and PFOA as the most prevalent pollutants, and the Niuchao lakelet was loaded with the greatest PFC pollutions attributed to more human activities. The concentrations of PFUnDA, PFDoDA and PFTA were lower than LOQs in most (>70%) of collected water samples, and the possible reason was that the long-chain (C > 8) PFCs are potential to accumulate in sediment and aquatic biota. Herein, for the future studies, a comprehensive and multiple dimensional survey of PFC contamination for East Lake is highly required.

## Methods

### Chemicals and reagents

The nine PFCs, including PFHxS (purity 98%), PFOS (98%), PFHpA (99%), PFOA (96%), PFNA (97%), PFDA (98%), PFUnDA (96%), PFDoDA (95%) and PFTA (97%), were purchased from Sigma-Aldrich Company (St. Louis, MO, USA). Perfluoro-1-[1,2,3,4-^13^C_4_]octane sulfonate (MPFOS, 98%) and perfluoro-n-[1,2,3,4-^13^C_4_]octanoic acid (MPFOA, 98%) were bought from Wellington Laboratories (Guelph, Ontario, Canada), with an initial concentration at 50 ± 2.5 μg mL^−1^ level in methanol. Multi-PFCs stock solution was prepared in methanol: water (7: 3, v/v) with a final concentration of 0.1 μg mL^−1^ for each analyte, and the IS solution was also prepared at 0.5 μg mL^−1^ level. The stock solutions were stored in a refrigerator at 4 °C and replaced every two weeks.

Methanol, acetonitrile and ammonium acetate were all of HPLC grade used in this study, supplied by Merck KGaA (Darmstadt, Germany). Ammonia-methanol solution (7 mol L^−1^) and HCl (35–38 wt%) were provided by Meryer Chemical Technology Co., Ltd. (Shanghai, China). The prepared Fe_3_O_4_@SiO_2_-NH_2_&F_13_ was rinsed thoroughly with acetonitrile: ammonia-methanol solution (6: 4, v/v) for several times and then dispersed in ethanol: water (5: 5, v/v) to form a stable colloid solution with a concentration of 25 mg mL^−1^. The threefold distilled water was used throughout the whole experiment.

### Water sample collection

In this study, 17 typical water sampling sites were selected for East Lake from 3 main lakelets (Guozheng, Niuchao and Tangling) along the lakeshore (Fig. 2), and the sampling sites were located by global position system (GPS). In each site, three parallel water samples were collected at a depth of 10 cm under the water surface and loaded in a 1 L of PP bottle, respectively. The PP bottle was rinsed meticulously according to the method recommended by US EPA^39^. To eliminate the contamination derived from the used materials, all of the accessible polytetrafluoroethene (PTFE) and fluoropolymer were avoid seriously during the sample collection and preparation. All samples were adjusted to pH = 3 with HCl (35–38 wt%) and frozen at −20 °C until analysis.

### Water sample preparation

**Figure 4:**
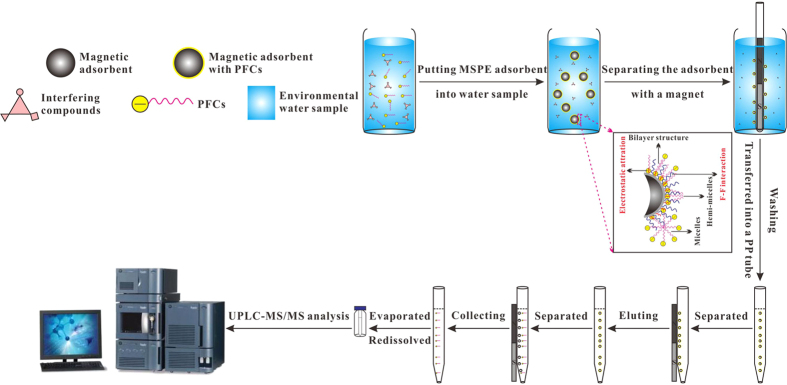
Schematic illustration of MSPE procedure and UPLC-MS/MS detection for PFCs in surface water sample from East Lake.

The frozen water samples were placed at room temperature until completely melted. The three parallel samples collected in each sampling site were all prepared by the optimized MSPE procedure. Typically, each water sample was treated by ultrasonic for 10 min and filtered through a disposable polyethersulfone membrane (0.45 μm). A volume of 500 mL of the water sample was spiked with 5 ng L^−1^ of ISs and then extracted by 50 mg of Fe_3_O_4_@SiO_2_-NH_2_&F_13_ with the help of a sonicate (200 W) for 5 min. After 30 min of sorption, a glass tube with a bar magnet embedded in the inner cavity was inserted into the water, and Fe_3_O_4_@SiO_2_-NH_2_&F_13_ was adsorbed onto the ektexine of the tube in 5 min. As shown in Fig. 4, the magnetic material was transferred into a 15 mL of PP tube and washed with 10 mL of methanol: water (3: 7, v/v) for 2 min. The target analytes were eluted by 10 mL of acetonitrile: ammonia-methanol solution (7: 3, v/v) in 2 min. The elution solution was collected into another PP tube and evaporated at 40 °C under a gentle flow of N_2_. The residue was redissolved with 0.5 mL of methanol: water (7: 3, v/v) and filtered with a nylon membrane (0.22 μm) for further UPLC-MS/MS analysis, and the average value was adopted for each sampling site.

A volume of 1 L of the water sample collected in each sampling site was mixed with each other in a PP bucket, giving rise to a total water volume of about 17 L. The mixed water sample was spiked with 0.5, 5 and 50 ng L^−1^ of PFCs, respectively, and also prepared by the optimized MSPE procedure and used for method accuracy and precision evaluation. For comparison, the mixed water sample was also treated by BS ISO 25101:2009 standard method^31^ with Oasis-Wax (150 mg, 6 cm^3^) (Waters Corporation, Milford, MA, USA) as SPE adsorbent.

### UPLC-MS/MS analysis

An UPLC-MS/MS system (Waters Corporation) was used for separation and detection of nine PFCs analyzed in this study. The separation units were equipped with an Acquity^TM^ Binary Solvent Pump and a Sample Manager, coupled with an Agilent Zorbax Eclipse Plus C_18_ column (150 × 2.1 mm i.d., 3.5 μm) (Palo Alto, CA, USA). The mobile phase comprised two parts, A: 2 mmol L^−1^ ammonium acetate and B: methanol, with a flow rate of 0.2 mL min^−1^. The gradient elution was carried out as follows: 0–2 min, 30–75% B; 2–7.5 min, 75–95% B; 7.5–11 min, 95–100% B; 11–16 min, 100% B; 16–17 min, 100–30% B; 17–20 min, 30% B. The column temperature was kept at 30 °C and the injection volume was set at 10 μL. After each sample injection, the sampling needle was rinsed according to the UPLC needle-wash procedure, with acetonitrile (800 μL) and acetonitrile: water (2: 8, v/v) (500 μL) as strong and weak washing solvent, respectively.

A Xevo-TQD Triple Quadrupole Tandem Mass Spectrometry was used for PFC detection, and the analytes were negatively ionized by an electrospray ionization (ESI) source. Chromatograms were recorded in multiple reaction monitoring (MRM) mode with a dwell time of 50 ms. The ESI-MS/MS parameters (Table 6) were optimized using 1 μg mL^−1^ of PFC solution in methanol. The instrument operation and data processing were performed with Masslynx V4.1 software.

## Additional Information

**How to cite this article**: Zhou, Y. *et al*. Occurrence investigation of perfluorinated compounds in surface water from East Lake (Wuhan, China) upon rapid and selective magnetic solid-phase extraction. *Sci. Rep.* **6**, 38633; doi: 10.1038/srep38633 (2016).

**Publisher's note:** Springer Nature remains neutral with regard to jurisdictional claims in published maps and institutional affiliations.

## Figures and Tables

**Table 1:** Analytical parameters of the developed MSPE-UPLC-MS/MS method.

Analytes	Linearity ranges (ng L^−1^)	Calibration curves	Determination coefficients (R^2^)	Limits of quantification (LOQs) (ng L^−1^)	Limits of detection (LODs) (ng L^−1^)
PFHxS	0.5–100	Y = 0.7956X + 0.099	0.9983	0.102	0.031
PFOS	0.5–100	Y = 0.7526X + 0.035	0.9994	0.097	0.029
PFHpA	0.5–100	Y = 0.4705X + 0.372	0.9986	0.330	0.099
PFOA	0.5–100	Y = 0.8050X + 0.882	0.9963	0.208	0.062
PFNA	0.5–100	Y = 1.1559X + 0.322	0.9987	0.167	0.050
PFDA	0.5–100	Y = 1.0942X + 0.023	0.9979	0.165	0.049
PFUnDA	0.5–100	Y = 0.9997X + 0.202	0.9963	0.322	0.097
PFDoDA	0.5–100	Y = 1.1337X + 0.280	0.9959	0.211	0.063
PFTA	0.5–100	Y = 0.5286X + 0.271	0.9917	0.189	0.057

**Table 2:** Spiked recoveries of PFCs in the mixed water sample extracted by Fe_3_O_4_@SiO_2_-NH_2_&F_13_ and Oasis-Wax, respectively.

Analytes	Spiked recoveries (Average ± Standard deviation (%), n = 3) at three fortified concentrations (ng L^−1^)					
	Fe_3_O_4_@SiO_2_-NH_2_&F_13_^a^			Oasis-Wax^b^		
	0.5	5	50	0.5	5	50
PFHxS	104.06 ± 10.58	95.73 ± 9.21	101.40 ± 8.72	102.89 ± 9.56	107.91 ± 7.42	94.26 ± 4.10
PFOS	102.39 ± 10.58	102.47 ± 5.22	103.68 ± 3.23	94.32 ± 7.54	93.47 ± 7.19	97.21 ± 2.27
PFHpA	90.27 ± 8.99	99.40 ± 5.66	92.93 ± 8.61	no data^c^	87.97 ± 5.85	94.08 ± 3.92
PFOA	106.67 ± 10.55	101.87 ± 9.02	100.53 ± 9.13	86.75 ± 6.12	90.40 ± 9.21	102.78 ± 4.69
PFNA	90.05 ± 9.64	93.05 ± 4.76	92.55 ± 5.84	84.48 ± 8.43	109.88 ± 8.45	106.66 ± 7.47
PFDA	96.23 ± 12.11	104.83 ± 9.88	92.85 ± 4.51	79.82 ± 9.19	115.07 ± 8.78	105.01 ± 5.32
PFUnDA	98.53 ± 11.36	104.80 ± 6.09	99.27 ± 8.78	73.43 ± 6.91	97.82 ± 6.44	105.92 ± 3.17
PFDoDA	93.51 ± 8.76	90.16 ± 5.28	93.97 ± 6.73	69.08 ± 6.87	70.48 ± 1.61	86.37 ± 4.23
PFTA	99.51 ± 10.75	92.63 ± 3.25	99.94 ± 9.70	63.74 ± 8.16	70.21 ± 3.35	76.88 ± 1.79

^a^Used as MSPE adsorbent in this study.

^b^Used as SPE adsorbent recommended by BS ISO 25101:2009 standard method^6^.

^c^Response signal of PFHpA in spiked water sample equal to or lower than that in unspiked sample.

**Table 3:** Comparison of the analytical performance of the proposed MSPE-UPLC-MS/MS method with other ones for determination of PFCs in surface water sample.

Analytical methods	Adsorbents	Sorbent amount (mg)	Sample volume (mL)	Enrichment factor	Extraction time (min)	Analytical time^a^ (min)	Linearity ranges (ng L^−1^)	LODs (ng L^−1^)	Spike recoveries (%)	RSDs (%)
MSPE-UPLC-MS/MS^25^	Fe_3_O_4_@SiO_2_-FBC	40	100	100	12	3.5	0.25–25	0.01–0.06	89.34–111.32	<9.9
MSPE-HPLC-MS/MS^19^	Fe_3_O_4_-C_18_@Chitosan	100	500	1000	20	6.5	0.5–50	0.05–0.24	56–112	2–8
MSPE-HPLC-MS/MS^17^	Fe_3_O_4_-CTAB	75	800	1600	10	6.5	0.5–50	0.03–0.88	66–123	1–8
SPE-HPLC-MS/MS^13^	Oasis-Wax	150	1000	1000	>300	>10	0.2–50	<0.1–0.6^b^	61–118	<18
SPE-HPLC-MS/MS^15^	Oasis-HLB	200	500	500	>160	>20	n. r.^c^	<0.2–2.0^b^	77–137	n. r.^c^
This work	Fe_3_O_4_@SiO_2_-NH_2_&F_13_	50	500	1000	30	8	0.5–100	0.029–0.099	90.05–106.67	3.1–12.6

^a^Confirmed by the time of chromatogram peak appearance of the last target analyte.

^b^LOQs. The LODs were not given in the References.

^c^Not reported.

**Table 4:** Concentrations of PFCs in surface water from East Lake.

Sampling sites	PFC concentrations (ng L^−1^)									
	PFHxS	PFOS	PFHpA	PFOA	PFNA	PFDA	PFUnDA	PFDoDA	PFTA	ΣPFCs
G1	2.97	6.88	4.19	17.89	0.88	<LOQ	<LOQ	<LOQ	<LOQ	32.95
G2	2.85	6.95	7.02	21.51	1.08	0.29	<LOQ	<LOQ	0.35	40.30
G3	1.92	10.12	8.40	22.90	1.77	0.75	0.52	0.81	0.52	47.71
G4	3.04	6.32	2.97	17.20	0.56	0.19	<LOQ	<LOQ	<LOQ	30.28
G5	2.80	15.12	7.29	25.00	2.09	1.05	0.44	<LOQ	<LOQ	54.14
G6	1.61	12.22	5.82	21.38	0.76	0.25	<LOQ	0.49	0.81	43.41
G7	1.94	15.85	6.73	22.50	1.49	0.47	<LOQ	0.27	<LOQ	49.55
G8	1.32	10.82	5.03	23.35	0.63	0.19	<LOQ	<LOQ	<LOQ	41.34
N1	2.54	31.06	5.87	26.39	1.19	0.42	<LOQ	<LOQ	0.25	67.72
N2	2.17	21.54	5.67	20.66	1.09	<LOQ	<LOQ	<LOQ	<LOQ	51.26
N3	1.76	28.82	8.46	46.56	1.30	0.47	<LOQ	<LOQ	<LOQ	87.74
N4	0.48	4.59	4.96	19.40	1.08	0.40	<LOQ	<LOQ	<LOQ	30.91
N5	2.08	29.20	8.55	83.32	1.36	0.51	<LOQ	<LOQ	<LOQ	125.35
N6	2.24	24.80	7.15	20.56	1.12	0.22	<LOQ	<LOQ	<LOQ	56.15
T1	0.68	5.98	3.97	18.69	0.55	0.24	<LOQ	<LOQ	<LOQ	30.12
T2	1.23	11.18	5.01	22.56	1.10	0.29	<LOQ	<LOQ	<LOQ	41.37
T3	0.77	10.59	5.60	20.40	0.89	0.64	<LOQ	0.40	0.25	39.77

<LOQ: Less than the limit of quantification.

**Table 5:** Correlation of PFC analytes and ΣPFCs based on nonparametric tests.

		PFHxS	PFOS	PFHpA	PFOA	PFNA	PFDA	PFUnDA	PFDoDA	PFTA	ΣPFCs
PFHxS	Kendall’s tau.	1									
	*p* value	—									
PFOS	Kendall’s tau.	0.176	1								
	*p* value	0.323	—								
PFHpA	Kendall’s tau.	0.162	0.515	1							
	*p* value	0.365	0.004**	—							
PFOA	Kendall’s tau.	0.000	0.500	0.632	1						
	*p* value	1.000	0.005**	0.000**	—						
PFNA	Kendall’s tau.	0.111	0.435	0.627	0.524	1					
	*p* value	0.536	0.015*	0.000**	0.003**	—					
PFDA	Kendall’s tau.	−0.112	0.141	0.439	0.439	0.552	1				
	*p* value	0.536	0.433	0.015*	0.015*	0.002**	—				
PFUnDA	Kendall’s tau.	0.033	0.198	0.594	0.363	0.555	0.684	1			
	*p* value	0.861	0.294	0.002**	0.055	0.003**	0.000**	—			
PFDoDA	Kendall’s tau.	−0.066	0.132	0.462	0.231	0.389	0.551	0.815	1		
	*p* value	0.727	0.485	0.014*	0.221	0.040*	0.004**	0.000**	—		
PFTA	Kendall’s tau.	−0.025	0.041	0.274	0.174	0.150	0.411	0.447	0.595	1	
	*p* value	0.896	0.827	0.149	0.358	0.431	0.032*	0.026*	0.003**	—	
ΣPFCs	Kendall’s tau.	0.221	0.838	0.676	0.632	0.598	0.275	0.314	0.215	0.075	1
	*p* value	0.217	0.000**	0.000**	0.000**	0.001**	0.127	0.097	0.256	0.694	—

^*^p < 0.05, ^**^p < 0.01.

**Table 6:** Optimized parameters of ESI-MS/MS for PFCs and the corresponding IS surrogates.

Analytes	Parent ion (m/z)	Daughter ion^a^ (m/z)	Cone (V)	Collision energy (V)
PFHxS	398.8	79.9/98.9	60	35/25
PFOS	498.8	79.9/98.9	55	35/30
PFHpA	362.8	318.9/169.0	10	5/15
PFOA	412.8	369.0/169.0	15	5/15
PFNA	462.8	419.0/218.9	15	10/15
PFDA	512.8	468.9/219.0	15	10/15
PFUnDA	562.8	518.9/269.0	15	10/20
PFDoDA	612.8	568.9/269.0	15	10/20
PFTA	712.8	669.0/169.0	15	10/25
MPFOS	502.9	80.0/99.0	55	40/30
MPFOA	416.9	372.0/169.0	15	5/15

^a^First ion was used as the quantifier and the second as the qualifier.
